# Antihyperalgesic Activity of Atomoxetine on Diabetes-Induced Neuropathic Pain: Contribution of Noradrenergic and Dopaminergic Systems

**DOI:** 10.3390/molecules23082072

**Published:** 2018-08-19

**Authors:** Mustafa Burak Barbaros, Özgür Devrim Can, Umut İrfan Üçel, Nazlı Turan Yücel, Ümide Demir Özkay

**Affiliations:** Department of Pharmacology, Faculty of Pharmacy, Anadolu University, 26470 Eskişehir, Turkey; mburakbarbaros@gmail.com (M.B.B.); uiucel@anadolu.edu.tr (U.İ.Ü.); nazlituran@anadolu.edu.tr (N.T.Y.); udemir@anadolu.edu.tr (Ü.D.Ö.)

**Keywords:** atomoxetine, catecholamine, Hargreaves test, hyperalgesia, neuropathy, Randall-Selitto test

## Abstract

Atomoxetine is a selective noradrenaline reuptake inhibitor drug. Based on the knowledge that agents increasing monoamine levels in the central nervous system have therapeutic potential for neuropathic pain, it is planned to investigate the possible efficacy of atomoxetine on diabetes-induced hyperalgesia, in this study. Randall-Selitto (mechanical noxious stimuli) and Hargreaves (thermal noxious stimuli) tests were used to evaluate nociceptive perception of rats. Obtained data indicated that streptozotocin-induced diabetes causes significant decreases in the paw withdrawal threshold and paw withdrawal latency values of the animals, respectively. However, atomoxetine administered at 3 mg/kg/day for 7 and 14 days improved these diabetes-induced hyperalgesia responses. Furthermore, antihyperalgesic activity was antagonized with α-methyl-para-tyrosine methyl ester, phentolamine, propranolol, and sulpiride pre-treatments. The same effect was not reversed, however, by SCH 23390. These findings demonstrated, for the first time, that atomoxetine possesses significant antihyperalgesic activity on diabetes-induced neuropathic pain and this effect seems to be mediated by α- and β-adrenergic and D_2_/D_3_ dopaminergic receptors. Results of this present study seem to offer a new indication for an old drug; atomoxetine, but these preclinical data should first be confirmed with further well-designed clinical trials.

## 1. Introduction

Chronic exposure to hyperglycemia is known to cause serious damage to the nervous system of patients with diabetes mellitus. This damage, which is directly induced by diabetes itself, but not related to any other inherited, traumatic, nutritional, metabolic, infectious, immunological, neoplastic, or toxic reasons, is called diabetic neuropathy (DN). Therefore, DN may simply be defined as the presence of peripheral nerve dysfunction-related symptoms in diabetic patients, after the exclusion of other possible reasons for neuropathy [1,2].

Diabetic neuropathy, as one of the long-term complications of diabetes, eventually develops in up to 50% of the patients and severely restricts their daily life [1]. The most common type of diabetic neuropathy is chronic sensorimotor peripheral polyneuropathy [3]. The clinical appearances of DN alter depending on the type of affected nerve fibres; sensory, motor, or autonomic. DN-induced damage to sensory neurons can cause either a “painful condition” characterized by allodynia (pain perception in response to non-painful stimuli) and hyperalgesia (exaggerated pain sensations to painful stimuli) or a “painless syndrome”, with disappearance of sensation to pain, heat, or touch stimulus [4]. Moreover, difficulties in some motor behaviours, such as handling small objects or climbing stairs, can be observed in patients with motor fibre involvement [3]. 

Providing a strict glycemic control, normalization of blood pressure level, education of diabetic patients and encouraging proper lifestyle changes are the basic treatment strategies valid for all types and stages of neuropathy [5]. There are also some alternatives for pathogenetic treatment of DN. On the other hand, main pharmacotherapy options for the symptomatic treatment of DN-induced pain can be listed as antidepressants (such as amitriptyline, clomipramine, imipramine, duloxetine), anticonvulsants (such as carbamazepine, gabapentin, pregabalin) and opioid drugs (such as tramadol, oxycodone) as well as some topical agents (such as capsaicin cream or lidocaine patches) [6]. However, clinically, treating painful diabetic neuropathy is extremely difficult and the majority of patients do not, or only partially, respond to these mentioned treatments. Therefore, development of potent drugs for relieving chronic neuropathic pain in diabetic patients has a notable clinical significance.

Atomoxetine (Strattera^®^) is the first non-stimulant drug approved by FDA for the management of attention deficit hyperactivity disorder (ADHD). It is classified as a “selective noradrenaline reuptake inhibitor” and its mechanism of action has been associated with the enhancement in the levels of intra-synaptic noradrenaline in the central nervous system. This drug is also shown to increase the extracellular levels of dopamine in the prefrontal cortex, via inhibition of the noradrenaline transporter. Different from psychostimulant drugs, absence of the abuse potency is an important advantage for atomoxetine. On the other hand, in ADHD treatment, late onset of the therapeutic action, as well as its lower efficacy comparing to the psychostimulants, can be listed as main disadvantages of this drug. Decreased appetite, headache, abdominal pain, xerostomia, hyperhidrosis, insomnia, drowsiness, nausea, vomiting, constipation, and erectile dysfunction are among the main adverse effects of atomoxetine. In addition, health professionals should be aware of the comorbid liver failure and increased suicidal ideations in patients taking this drug [7].

It is well described that noradrenaline plays an important role in the pain processing and analgesia. Moreover, recent studies pointed out that enhanced level of noradrenaline in the spinal and supraspinal pathways is crucial for the analgesic effects of various drugs on neuropathic pain [8,9,10,11]. Thus, drugs increasing noradrenaline levels in the central nervous system may have a therapeutic potential for patients suffering from the neuropathic pain disorders. In this context, it can be hypothesized that atomoxetine, as a selective noradrenaline reuptake inhibitory drug, may be effective in the treatment of neuropathic pain. Therefore, in this study, it was planned to investigate promising efficacy of atomoxetine on mechanical and thermal hyperalgesia developing in diabetic rats, and to elucidate the possible pharmacological mechanisms underlying the effect.

## 2. Results

### 2.1. Beneficial Effects of Atomoxetine on Neuropathic Pain

#### 2.1.1. Atomoxetine Alleviates Diabetes-Induced Mechanical Hyperalgesia

**Figure 1:**
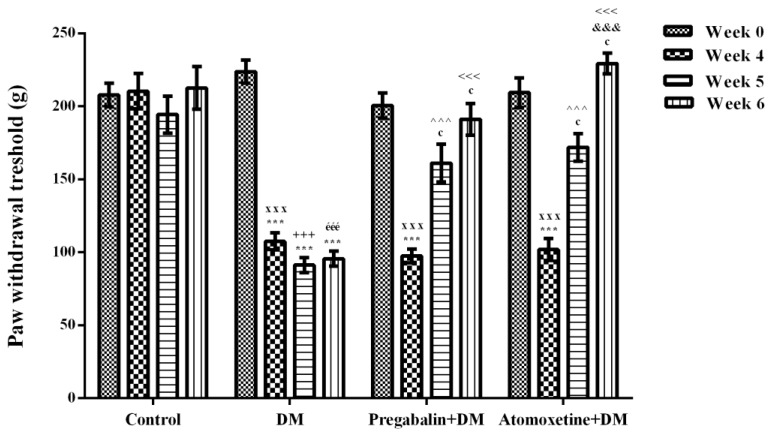
Paw withdrawal thresholds of normoglycemic rats administrated physiological saline (Control), and of diabetic rats administrated daily 10 mg/kg pregabalin (Pregabalin + DM) and daily 3 mg/kg atomoxetine (Atomoxetine + DM), in the Randall-Selitto test. Values are given as mean ± S.E.M. Within the groups: significant difference against Week 0, Week 4 and Week 5 groups are *** *p* < 0.001; ^c^ *p* < 0.001 and ^&&&^ *p* < 0.001, respectively. Between the groups: significant difference against Week 4 control, Week 5 control, Week 6 control, Week 5 DM and Week 6 DM groups are ^xxx^ *p* < 0.001; ^+++^ *p* < 0.001, ^ééé^ *p* < 0.001, ^^^^^ *p* < 0.001 and ^<<<^ *p* < 0.001, respectively. Two-way repeated ANOVA, post hoc Bonferroni test, *n* = 8.

Figure 1 presents the effect of atomoxetine (3 mg/kg/day, 2 weeks) treatment on mechanical nociceptive stimulus-induced hyperalgesia responses in diabetic rats. Two-way repeated ANOVA analysis showed that both of the treatment [F (3,28) = 38.73, *p* < 0.001] and the time factors [F (3,84) = 59.21, *p* < 0.001] were effective on the paw withdrawal thresholds of rats measured in the Randall-Selitto tests. Besides, there was a significant interaction between the “treatment” and the “time” factors [F (9,84) = 18.14, *p* < 0.001]. Results acquired from the Bonferroni multiple comparison tests demonstrated that in all of the diabetic groups “paw withdrawal threshold” values of rats measured at 4th weeks were significantly lower than those measured before the induction of diabetes (Week 0) (Figure 1.). On the other hand, administration of atomoxetine at doses of 3 mg/kg for 7 (*p* < 0.001) and 14 (*p* < 0.001) days significantly prolonged the reduced paw withdrawal thresholds of diabetic rats. In addition, 14-day administrations of atomoxetine were found to be more effective than the 7-day administrations (*p* < 0.001). Pregabalin also showed the expected antihyperalgesic efficacy following the 7 (*p* < 0.001) and 14 (*p* < 0.001) day-long treatments (Figure 1).

#### 2.1.2. Atomoxetine Alleviates Diabetes-Induced Thermal Hyperalgesia 

**Figure 2:**
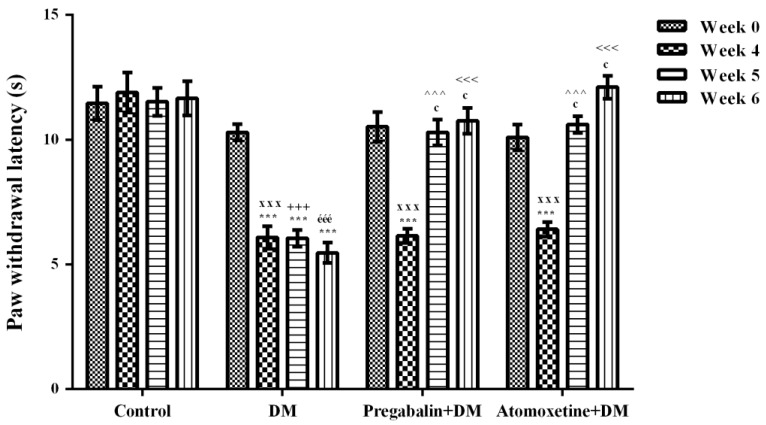
Paw withdrawal latency of normoglycemic rats administrated physiological saline (Control), and of diabetic rats administrated daily 10 mg/kg pregabalin (Pregabalin + DM) and daily 3 mg/kg atomoxetine (Atomoxetine + DM), in the in the Hargreaves test. Values are given as mean ± S.E.M. Within the groups: significant difference against Week 0 and Week 4 groups are *** *p* < 0.001 and ^c^ *p* < 0.001, respectively. Between the groups: significant difference against Week 4 control, Week 5 control, Week 6 control, Week 5 DM and Week 6 DM groups are ^xxx^ *p* < 0.001; ^+++^ *p* < 0.001, ^ééé^ *p* < 0.001, ^^^^^ *p* < 0.001 and ^<<<^ *p* < 0.001, respectively. Two-way repeated ANOVA, post hoc Bonferroni test, *n* = 8.

The effect of atomoxetine treatment (3 mg/kg/day, 2 weeks) on thermal nociceptive stimulus-induced hyperalgesia responses in diabetic rats was shown in Figure 2. Two-way repeated ANOVA analysis showed that both the treatment [F (3,28) = 62.31, *p* < 0.001] and the time factors [F (3,84) = 25.07, *p* < 0.001] were effective on the paw withdrawal latency values of rats measured in the Hargreaves tests. Besides, there was a significant interaction between the “treatment” and the “time” factors [F (9,84) = 11.59, *p* < 0.001]. Results acquired from the Bonferroni multiple comparison tests demonstrated that in all of the diabetic experimental groups “paw withdrawal latency” values of rats measured at 4th weeks were significantly lower than those measured before the induction of diabetes (Week 0) (Figure 2). On the other hand, administration of atomoxetine at doses of 3 mg/kg for 7 (*p* < 0.001) and 14 (*p* < 0.001) days significantly prolonged the shortened paw withdrawal latency values of diabetic rats. In addition, pregabalin showed the expected antihyperalgesic efficacy following the 7 (*p* < 0.001) and 14 (*p* < 0.001) day-long treatments (Figure 2).

#### 2.1.3. Catecholaminergic System Mediates the Beneficial Effect of Atomoxetine on Diabetes-Induced Mechanical and Thermal Hyperalgesia

**Figure 3:**
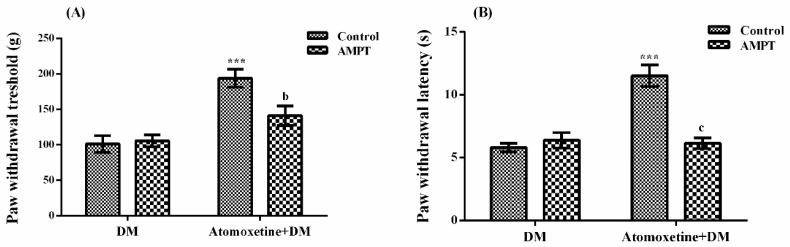
Effects of AMPT pre-treatments on the antihyperalgesic responses induced by atomoxetine (3 mg/kg, 14 days) against mechanical (**A**) and thermal (**B**) nociceptive stimuli applied in the Randall-Selitto and Hargreaves tests, respectively. Values are given as mean ± S.E.M. Significant difference against physiological saline administrated diabetic group (DM) is *** *p* < 0.001; Significant difference against atomoxetine administrated diabetic groups (Atomoxetine + DM) are ^b^ *p* < 0.01 and ^c^ *p* < 0.001. Two-way repeated ANOVA, post hoc Bonferroni test, *n* = 8.

The effect of α-methyl-para-tyrosine methyl ester (AMPT) pre-treatment on atomoxetine-induced antihyperalgesic response against mechanical nociceptive stimuli applied in the Randall-Selitto test was shown in Figure 3A. The results of two-way ANOVA analysis showed significant effects of both atomoxetine treatment [F (1,28) = 29.70, *p* < 0.001] and AMPT administration [F (1,28) = 4.22, *p* < 0.05] on the paw withdrawal thresholds of diabetic rats. In addition, a significant interaction was detected between the "atomoxetine treatment" and "AMPT administration" factors [F (1,28) = 5.94, *p* < 0.05). Results of the Bonferoni multiple comparisons test showed that AMPT administrations significantly reversed the atomoxetine-induced antihyperalgesic responses in the Randall-Selitto tests (*p* < 0.01). 

Figure 3B shows the effect of AMPT pre-treatment on atomoxetine-induced antihyperalgesic responses against thermal nociceptive stimuli applied in the Hargreaves test. The results of two-way ANOVA analysis showed significant effects of both atomoxetine treatment [F (1,28) = 21.45, *p* < 0.001] and AMPT administration [F (1,28) = 16.54, *p* < 0.001] on the paw withdrawal latencies of diabetic rats. In addition, a significant interaction was detected between the “atomoxetine treatment” and “AMPT administration” factors [F (1,28) = 25.24, *p* < 0.001]. Results of the Bonferoni multiple comparisons test showed that AMPT administrations significantly reversed the atomoxetine-induced antihyperalgesic responses assessed in the Hargreaves test (*p* < 0.001).

**Figure 4:**
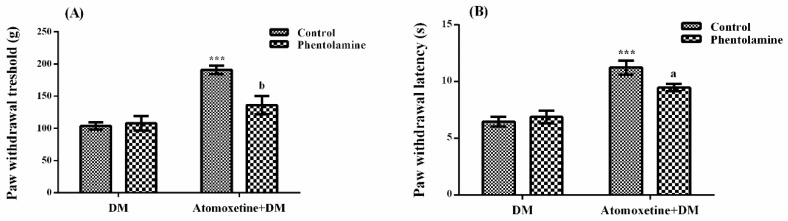
Effects of phentolamine pre-treatments on the antihyperalgesic responses induced by atomoxetine (3 mg/kg, 14 days) against mechanical (**A**) and thermal (**B**) nociceptive stimuli applied in the Randall-Selitto and Hargreaves tests, respectively. Values are given as mean ± S.E.M. Significant difference against physiological saline administrated diabetic group (DM) is *** *p* < 0.001; Significant difference against atomoxetine administrated diabetic groups (Atomoxetine+DM) are ^a^ *p* < 0.05 and ^b^ *p* < 0.01. Two-way repeated ANOVA, post hoc Bonferroni test, *n* = 8.

The effect of phentolamine (a non-selective α-adrenergic receptor blocker agent) pre-treatments on atomoxetine-induced antihyperalgesic response against mechanical nociceptive stimuli applied in the Randall-Selitto test was shown in Figure 4A. The results of two-way ANOVA analysis showed significant effects of both atomoxetine treatment [F (1,28) = 33.18, *p* < 0.001] and phentolamine administrations [F (1,28) = 6.34, *p* < 0.05] on the paw withdrawal thresholds of diabetic rats. In addition, a significant interaction was detected between the “atomoxetine treatment” and “phentolamine administration” factors [F (1,28) = 8.66, *p* < 0.01]. Results of the Bonferoni multiple comparisons test showed that phentolamine administration significantly reversed the atomoxetine-induced antihyperalgesic responses in the Randall-Selitto tests (*p* < 0.01).

Figure 4B presents the effect of phentolamine pre-treatment on atomoxetine-induced antihyperalgesic response against thermal nociceptive stimuli applied in the Hargreaves test. The results of the two-way ANOVA analysis showed that the effect of atomoxetine treatment on the paw withdrawal latencies of diabetic rats was significant [F (1,28) = 55.26, *p* < 0.001] but the effect of phentolamine was not [F (1,28) = 1.87, *p* > 0.05]. In addition, a significant interaction was detected between the “atomoxetine treatment” and “phentolamine administration” factors [F (1,28) = 4.84, *p* < 0.05]. Results of the Bonferoni multiple comparisons test showed that phentolamine administration significantly reversed the atomoxetine-induced antihyperalgesic responses assessed in the Hargreaves test (*p* < 0.05). 

**Figure 5:**
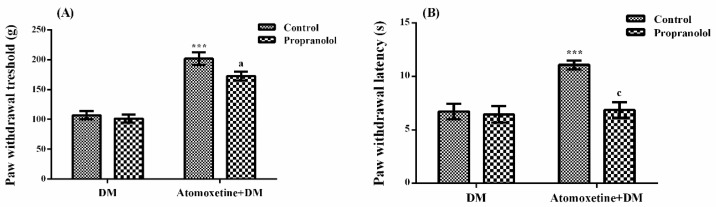
Effects of propranolol pre-treatments on the antihyperalgesic responses induced by atomoxetine (3 mg/kg, 14 days) against mechanical (**A**) and thermal (**B**) nociceptive stimuli applied in the Randall-Selitto and Hargreaves tests, respectively. Values are given as mean ± S.E.M. Significant difference against physiological saline administrated diabetic group (DM) is *** *p* < 0.001; Significant difference against atomoxetine administrated diabetic groups (Atomoxetine + DM) are ^a^ *p* < 0.05 and ^c^ *p* < 0.001. Two-way repeated ANOVA, post hoc Bonferroni test, *n* = 8.

The effect of propranolol (a non-selective β-adrenergic receptor blocker) pre-treatment on atomoxetine-induced antihyperalgesic response against mechanical nociceptive stimuli applied in the Randall-Selitto test was shown in Figure 5A. The results of two-way ANOVA analysis showed significant effects of both atomoxetine treatment [F (1,28) = 101.4, *p* < 0.001] and propranolol administrations [F (1,28) = 4.35, *p* < 0.05] on the paw withdrawal thresholds of diabetic rats. However, no significant interaction was detected between the “atomoxetine treatment” and the “propranolol administration” factors [F (1,28) = 2.06, *p* > 0.05]. Results of the Bonferoni multiple comparisons test showed that propranolol administration significantly reversed the atomoxetine-induced antihyperalgesic responses in the Randall-Selitto tests (*p* < 0.05).

Figure 5B displays the effect of propranolol pre-treatment on atomoxetine-induced antihyperalgesic response against thermal nociceptive stimuli applied in the Hargreaves test. The results of two-way ANOVA analysis showed significant effects of both atomoxetine treatment [F (1,28) = 12.49, *p* < 0.01] and propranolol administration [F (1,28) = 11.11, *p* < 0.01] on the paw withdrawal latencies of diabetic rats. In addition, a significant interaction was detected between the “atomoxetine treatment” and “propranolol administration” factors [F (1,28) = 8.55, *p* < 0.01]. Results of the Bonferoni multiple comparisons test showed that propranolol administration significantly reversed the atomoxetine-induced antihyperalgesic responses assessed in the Hargreaves test (*p* < 0.001).

**Figure 6:**
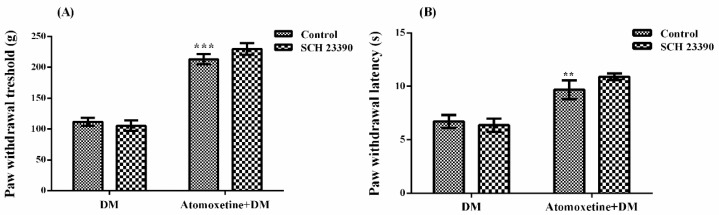
Effects of SCH 23390 pre-treatments on the antihyperalgesic responses induced by atomoxetine (3 mg/kg, 14 days) against mechanical (**A**) and thermal (**B**) nociceptive stimuli applied in the Randall-Selitto and Hargreaves tests, respectively. Values are given as mean ± S.E.M. Significant difference against physiological saline administrated diabetic group (DM) is ** *p* < 0.01; *** *p* < 0.001. Two-way repeated ANOVA, post hoc Bonferroni test, *n* = 8.

The effect of SCH 23390 (D_1_ dopaminergic receptor blocker) pre-treatment on atomoxetine-induced antihyperalgesic response against mechanical nociceptive stimuli applied in the Randall-Selitto test was shown in Figure 6A. The results of the two-way ANOVA analysis showed significant effect of atomoxetine treatment [F (1,28) = 190.1, *p* < 0.001] on the paw withdrawal thresholds of diabetic rats, but SCH 23390 administrations were ineffective [F (1,28) = 0.4, *p* > 0.05]. Besides, no significant interaction was detected between the “atomoxetine treatment” and the “SCH 23390 administration” factors [F (1,28) = 1.96, *p* > 0.05]. Results of the Bonferoni multiple comparisons test showed that SCH 23390 administration could not change the atomoxetine-induced antihyperalgesic responses in the Randall-Selitto tests (*p* > 0.05).

Figure 6B demonstrates the effect of SCH 23390 pre-treatment on atomoxetine-induced antihyperalgesic response against thermal nociceptive stimuli applied in the Hargreaves test. The results of the two-way ANOVA analysis showed significant effect of atomoxetine treatment [F (1,28) = 34.57, *p* < 0.001] on the paw withdrawal latencies of diabetic rats but, SCH 23390 administrations were ineffective [F (1,28) = 0.47, *p* > 0.05]. Besides, no significant interaction was detected between the “atomoxetine treatment” and the “SCH 23390 administration” factors [F (1,28) = 1.49, *p* > 0.05]. Results of the Bonferoni multiple comparisons test showed that SCH 23390 administration could not reverse the atomoxetine-induced antihyperalgesic responses assessed in the Hargreaves test (*p* > 0.05).

**Figure 7:**
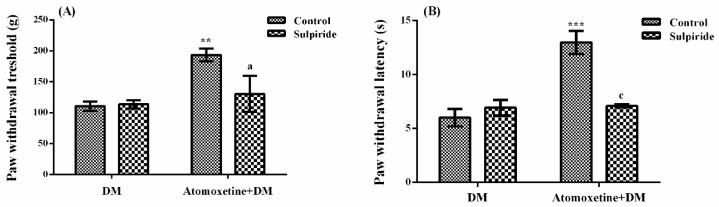
Effects of sulpiride pre-treatments on the antihyperalgesic responses induced by atomoxetine (3 mg/kg, 14 days) against mechanical (**A**) and thermal (**B**) nociceptive stimuli applied in the Randall-Selitto and Hargreaves tests, respectively. Values are given as mean ± S.E.M. Significant difference against physiological saline administrated diabetic group (DM) is ** *p* < 0.01; *** *p* < 0.001; Significant difference against atomoxetine administrated diabetic groups (Atomoxetine+DM) are ^a^ *p* < 0.05 and ^c^ *p* < 0.001. Two-way repeated ANOVA, post hoc Bonferroni test, *n* = 8.

The effect of sulpiride (D_2_/D_3_ dopaminergic receptor blocker) pre-treatment on atomoxetine-induced antihyperalgesic response against mechanical nociceptive stimuli applied in the Randall-Selitto test was shown in Figure 7A. The results of two-way ANOVA analysis showed significant effect of atomoxetine treatment [F (1,28) = 9.36, *p* < 0.01] on the paw withdrawal thresholds of diabetic rats but sulpiride administrations were ineffective [F (1,28) = 3.4, *p* > 0.05]. Besides, no significant interaction was detected between the “atomoxetine treatment” and “sulpiride administration” factors [F (1,28) = 4.12, *p* > 0.05]. Results of the Bonferoni multiple comparisons test showed that sulpiride administration significantly reversed the atomoxetine-induced antihyperalgesic responses in the Randall-Selitto tests (*p* < 0.05).

The effect of sulpiride pre-treatment on atomoxetine-induced antihyperalgesic response against thermal nociceptive stimuli applied in the Hargreaves test was shown in Figure 7B. The results of two-way ANOVA analysis showed significant effects of both atomoxetine treatment [F (1,28) = 22.07, *p* < 0.001] and sulpiride administration [F (1,28) = 10.60, *p* < 0.01] on the paw withdrawal latencies of diabetic rats. In addition, a significant interaction was detected between the “atomoxetine treatment” and "sulpiride administration" factors [F (1,28) = 20.0, *p* < 0.001]. Results of the Bonferoni multiple comparisons test showed that sulpiride administration significantly reversed the atomoxetine-induced antihyperalgesic responses assessed in the Hargreaves test (*p* < 0.001). 

### 2.2. Antihyperalgesic Efficacy of Atomoxetine is not Related to a Possible Alteration in the Motor Activity of Rats

**Figure 8:**
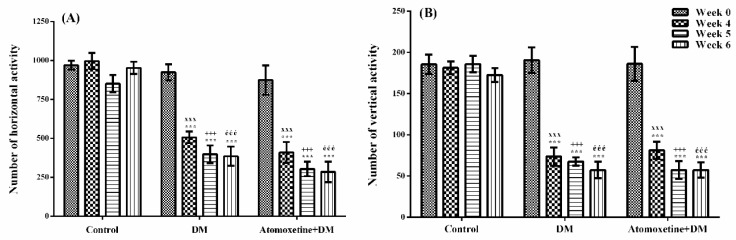
Total number of horizontal (**A**) and vertical (**B**) locomotor activities of normoglycemic rats administrated physiological saline (Control), and of diabetic rats administrated daily 3 mg/kg atomoxetine (Atomoxetine + DM), in the activity cage test. Values are given as mean ± S.E.M. Within the groups: significant difference against Week 0 group is *** *p* < 0.001. Between the groups: significant difference against Week 4 control, Week 5 control and Week 6 control groups are ^xxx^ *p* < 0.001; ^+++^ *p* < 0.001, and ^ééé^ *p* < 0.001, respectively. Two-way repeated ANOVA, post hoc Bonferroni test, *n* = 8.

The effects of atomoxetine on the horizontal and vertical locomotor activities of animals were shown in Figure 8A,B, respectively.

The results obtained from the two-way repeated ANOVA tests showed that both of the treatment [F (2,21) = 59.42, *p* < 0.001] and the time [F (3,63) = 35.31, *p* < 0.001] factors were effective on the number of horizontal locomotor activity, together with the statistically significant interaction between the “treatment” and the “time” factors [F (6,63) = 6.88, *p* < 0.001]. Furthermore, both of the treatment [F (2,21) = 55.29, *p* < 0.001] and the time [F (3,63) = 45.61, *p* < 0.001] factors were effective on the number of vertical locomotor activity, together with the statistically significant interaction between the “treatment” and the “time” factors [F (6,63) = 9.97, *p* < 0.001]. 

The results of the Bonferroni tests revealed that the number of horizontal and vertical locomotor activities measured at 4th week in all of the diabetic groups were significantly lower than those of measured before the induction of diabetes (Week 0) (*p* < 0.001). On the other hand, atomoxetine treatment did not induce a further change in the locomotor activities (Figure 8).

## 3. Discussion

Based on the curative activity potentials of monoamine reuptake inhibitory drugs on neuropathic pain [8,9,10,11,12,13], promising therapeutic efficacy of atomoxetine, a selective noradrenaline reuptake inhibitor drug, on diabetes-induced hyperalgesia was investigated, in the present study.

Diabetes was established with a single i.v. dose of streptozotocin (STZ). 4 weeks after the induction of diabetes, when neuropathy was developed in diabetic rats, hyperalgesia tests were started [12,13]. Two well-known experimental methods were used to assess antihyperalgesic activity of atomoxetine against mechanical and thermal painful stimuli: Randall-Selitto and Hargreaves tests, respectively. In both of these tests, in all of the diabetic experimental groups, “paw withdrawal threshold” (Figure 1) and “paw withdrawal latency” (Figure 2) values of rats measured at 4th weeks were significantly lower than those measured before the induction of diabetes (at week 0). These findings indicated that targeted neuropathy model was successfully established, in the present study.

Following the confirmation of the development of neuropathy at the 4^th^ week, diabetic rats were started to receive atomoxetine at daily dose of 3 mg/kg. Administrations of atomoxetine for 7 (*p* < 0.001) and 14 (*p* < 0.001) day-long induced a significant increase in the reduced paw withdrawal thresholds of diabetic rats in the Randall-Selitto tests (Figure 1.). Similarly, in the Hargreaves tests, shortened paw withdrawal latencies of diabetic rats were prolonged with the 7 (*p* < 0.001) and 14 (*p* < 0.001) day-long treatments with atomoxetine (Figure 2.). These data indicated that atomoxetine has a notable antihyperalgesic effect in diabetic rats, which is comparable with the efficacy of reference drug pregabalin.

After the antihyperalgesic effect of atomoxetine has been demonstrated, some possible mechanisms underlying this pharmacological effect were investigated by further studies. For this purpose, first, possible contribution of catecholaminergic system to the observed antihyperalgesic effect was investigated by using AMPT, an agent inhibiting the synthesis of catecholamines [14,15]. Obtained data indicated that AMPT pre-treatments reversed the atomoxetine-induced antihyperalgesic responses both in the Randall-Selitto (*p* < 0.01) and the Hargreaves tests (*p* < 0.001) (Figure 3).

Alpha-methyl-p-tyrosine is an agent selectively inhibiting tyrosine hydroxylase, a rate-limiting enzyme in the noradrenaline and dopamine synthesis [14,15]. Two consecutive injections of this agent at a dose of 200 mg/kg with 23 h interval, has been shown to cause a 50%–60% reduction in the central noradrenaline levels [14,16]. Therefore, in this study, abolishment of atomoxetine induced antihyperalgesic activity following AMPT pre-treatments suggested that catecholaminergic system could mediate the antihyperalgesic effect of atomoxetine, at least partially. Based on these findings and noradrenaline reuptake inhibitory mechanism of atomoxetine, it can be speculated that this drug might show its pharmacological effect on neuropathic pain by enhancing neurotransmission in the brain stem-spinal descending noradrenaline system, which suppresses nociceptive signaling from primary afferent neurons to the spinal dorsal horn [17]. Moreover, enhanced noradrenaline level in the dorsal horn of the spinal cord might induce antihyperalgesic activity by inhibiting the release of excitatory neurotransmitters from primary afferent fibers or by hyperpolarizing post-synaptic spinal cord dorsal horn cells, via activating spinal α-adrenergic receptors [11]. However, all these possible mechanisms should be clarified by further detailed studies. Actually, contribution of catecholaminergic system to the presented antihyperalgesic activity seem to get additional importance in terms of diabetic neuropathic pain treatment, since diabetes causes serious deficits in the functions of several neurotransmitter systems playing role in pain-suppressing pathways in the central nervous system, including noradrenergic system [18].

As a general knowledge, two main receptor subtypes are known to mediate the physiological effects of noradrenaline: α- and β-adrenoceptors. Both of the α-adrenergic [19,20,21] and β-adrenergic receptors [22,23] are known to play active roles in nociception and pain-related processes. Therefore, after demonstrating the participation of catecholaminergic system to the antihyperalgesic activity of atomoxetine, we examined the possible involvement of α- and β-adrenergic receptors in the mentioned activity of this drug. We used phentolamine, a non-selective α-adrenergic receptor blocker, and propranolol, a non-selective β-adrenergic receptor blocker, with this aim.

In the Randall-Selitto tests, pre-treatment with phentolamine significantly reversed atomoxetine-induced increase in the paw withdrawal thresholds of diabetic rats (*p* < 0.01). Moreover, prolonged paw withdrawal latencies of atomoxetine-administrated diabetic rats were significantly shortened in the Hargreaves tests (*p* < 0.05), following the phentolamine administrations (Figure 4.). Similar to results of the phentolamine studies, pre-treatment with propranolol also reversed the atomoxetine-induced increase in the paw withdrawal thresholds (*p* < 0.05) and reduced the prolonged paw withdrawal latencies of diabetic rats (*p* < 0.001) (Figure 5.), indicating the contribution of both of the α- and β-adrenoceptors to the anthyperalgesic effect of atomoxetine. These findings are in agreement with the results of the previous studies reporting the critical role of α-adrenergic [19,24,25,26,27,28] and β-adrenergic [12,13,29,30,31,32,33] receptors in the therapeutic effects of various drugs on neuropathic pain.

Recent studies indicated that dopaminergic system is also related to the nociceptive transmission in the central nervous system [34]. It has been shown that dopaminergic neurons project from the A11 region in the dorsal posterior hypothalamus innervate the dorsal horn of the medulla spinalis, a critical region related to transmission of painful stimuli [35,36]. Moreover, dopaminergic neurons in the ventral tegmental area (A10) have been demonstrated to release dopamine in the nucleus accumbens and enhance analgesic activity through D_2_-like receptors [37,38]. Based on these facts, we also investigated probable participation of dopaminergic receptors to the presented antihyperalgesic effect of atomoxetine. Possible involvement of D_1_ and D_2_/D_3_ dopaminergic receptors were examined by SCH 23390 and sulpiride pre-treatments, respectively. Obtained results showed that sulpiride administrations significantly reversed the atomoxetine-induced antihyperalgesic responses both in the Randall-Selitto (*p* < 0.05) (Figure 7A) and Hargreaves (*p* < 0.001) tests (Figure 7B). However, SCH 23390 administrations did not induce any alteration on the same effect (*p* > 0.05) (Figure 6A,B).

These findings, indicating the involvement of D_2_/D_3_, but not D_1_ dopaminergic receptors in the antihyperalgesic effect of atomoxetine, are in accordance with the results of the previous studies suggesting that D_2_-like receptors mediate the dopamine-induced blockage of nociceptive transmission at the dorsal horn [35,36,39]. Although atomoxetine has no direct effect on the dopamine transporter, dopaminergic systems might be indirectly involved in the antihyperalgesic effect this drug, similar to some other drugs inhibiting reuptake of monoamines such as tricyclic antidepressants or serotonin and noradrenaline reuptake inhibitors [11,39,40].

It is well described that drug-induced alterations in motor activities of the rodents may affect the results of the experiments performed to evaluate pain. Therefore, monitoring the motor activity of animals throughout the experiments has a critical importance. For this reason, we evaluated the spontaneous locomotor activities of rats that we used in the tests. Data obtained from the activity cage tests designated that diabetes caused significant decrease both in the horizontal (Figure 8A) and vertical (Figure 8B) locomotor activities. These findings supported the previous studies reporting the impaired motor activity and motor coordination in diabetic animals [41,42,43]. On the other hand, subacute administration of atomoxetine at doses of 3 mg/kg did not cause any further decrease or increase in the locomotor activity suggesting that antihyperalgesic efficacy of this drug is independent of any probable alteration in the motor activity of the animals. 

In this work, some important findings have been revealed about the pharmacological mechanisms underlying the antihyperalgesic effect of atomoxetine, however, it is also possible to conduct further studies to elucidate the exact mechanism of action. For example, specific α- and β-adrenergic receptor subtypes contributing to the observed effect can be clarified by using specific α- and β-adrenergic receptor subtype blocker agents such as prazosin (α_1_-adrenergic receptor antagonists) [27]; idazoxan, yohimbine (α_2_-adrenergic receptor antagonists) [24,27] or ICI 118,551 (β_2_ adrenoceptor antagonist) [44]. Moreover, it will also be useful to investigate possible participation of other neurotransmitter systems associated with nociception and analgesia, such as opioidergic, glutaminergic, nitrergic, GABAergic, or muscarinic systems to the atomoxetine-induced antihyperalgesic effect. Furthermore, investigation of atomoxetine-induced alterations in central and peripheral nervous system at molecular levels, by using immunohistochemical and morphometric methods, may also help to produce new and valuable data related to mode of the presented antihyperalgesic action.

In summary, this is the first study presenting the significant antihyperalgesic activity capacity of atomoxetine against diabetes-induced neuropathic pain and suggesting the involvement of α- and β-adrenergic receptors and D_2_/D_3_ dopaminergic receptors as underlying pharmacological mechanisms. These results have pharmacological importance since they pointed out a possibility of a new indication for an old drug: atomoxetine. Nevertheless, atomoxetine may be regarded as an alternative drug in the symptomatic treatment of neuropathic pain only if the experimental findings of this preclinical study could be confirmed by well-designed clinical trials.

## 4. Materials and Methods

### 4.1. Animals

Experiments were performed using male Sprague-Dawley rats weighing 250–300 g at the same age. Rats were housed in well-ventilated rooms at 24 ± 1 °C in a 12-h light/12-h dark cycle where the lights were kept open between 8:00–20:00. The experimental procedure of this research was approved by the Anadolu University Animal Experiments Local Ethics Committee (Date: 15 December 2017 and Project code: 2017-04).

### 4.2. Chemicals

Atomoxetine hydrochloride (Strattera, Eli Lilly, IN, USA), STZ, phentolamine, pregabalin, AMPT, propranolol, SCH 23390, and sulpiride used in this study were purchased from Sigma-Aldrich (St. Louis, MO, USA) whereas trisodium citrate and citric acid were acquired from Merck (Darmstadt, Germany). Besides, physiological saline was obtained from Adeka (Samsun, Turkey). 

### 4.3. Induction of Experimental Diabetes Model in Rats

Animal model of diabetes was established by a single intravenous (i.v.) injection of STZ (50 mg/kg, adjusted to pH 4.5 in 0.1 M citrate buffer) into the tail vein of the rats, as described earlier [12]. An equal volume of citrate buffer was administrated (i.v.) to all healthy rats placed in control groups. 72 h later the injection of STZ, measurements of blood glucose were performed by using a Glukotrend^®^ device (Roche, Basel, Switzerland). Rats, whose blood glucose levels were higher than 300 mg/dL, were classified as diabetic.

### 4.4. Design of the Experimental Groups

Experimental groups (*n* = 8) for neuropathic pain experiments were designed as follows (Table 1).

Atomoxetine and pregabalin were administrated at every morning 9:00 a.m. for 2 weeks. In the test days, experiments were performed 30 min after the last i.p. dose of atomoxetine and 60 min after the last oral dose of pregabalin.

### 4.5. Neuropathic Pain Tests

After waiting 4 weeks for the appearance of peripheral neuropathy in diabetic animals [48], hyperalgesia responses were assessed by Randall-Selitto and Hargreaves tests.

#### 4.5.1. Randall-Selitto Test

The mechanical nociceptive stimulus-induced hyperalgesia was assessed by Randall-Selitto device (Ugo-basile, 37215, Verase, Italy) which applies increasing pressure on the dorsal parts of the hindpaws. The threshold of mechanical hyperalgesia was accepted as the force (grams) at which the rat withdrew its paw from the device. The cut off value was determined as 250 g in order to protect the paw from a possible tissue damage [12,13,49].

#### 4.5.2. Hargreaves Test (Plantar Test)

The thermal nociceptive stimulus-induced hyperalgesia was assessed by Hargreaves test device (Ugo-basile, 37370, Verase, Italy), which focuses radiant heat to hind-paws of the rats. The parameter for thermal hyperalgesia was accepted as the paw withdrawal latency (s) of rats, which means the time until the rat withdraws its paw from the hot stimulus. The response time was calculated by taking the average of the three measurements performed at 5 min intervals. The cut off time was determined as 20 s in order to protect the paw from a possible tissue damage [12,50].

#### 4.5.3. Mechanistic studies

Potential participation of catecholaminergic neurotransmission to the antihyperalgesic activity of atomoxetine was examined by AMPT (a catecholamine synthesis inhibitor) pre-treatment studies. For this purpose, AMPT was administrated at dose of 200 mg/kg i.p., twice at 24 h and 1 h before the last dose of atomoxetine [12,13,51].

Furthermore, possible contributions of α-adrenergic, β-adrenergic, D_1_ dopaminergic and D_2_/D_3_ dopaminergic receptors to the antihyperalgesic effect of atomoxetine were investigated by the pre-treatments of phentolamine (non-selective α-adrenergic receptor antagonist, 5 mg/kg, i.p.), propranolol (non-selective β-adrenergic receptor antagonist, 5 mg/kg, i.p), SCH 23390 (dopaminergic D_1_ receptor antagonist, 0.5 mg/kg, i.p.) and sulpride (dopaminergic D_2_/D_3_ receptor antagonist, 30 mg/kg, i.p.), respectively [12,13,52,53]. These specific receptor blockers were administrated 30 min before the last dose of atomoxetine [12,13].

Similar studies in the literature, as well as the previous studies in our laboratory, have been used to determine the doses of antagonists used in this study.

### 4.6. Activity Cage Tests

An activity cage device (Ugo-basile, 7420, Verase, Italy), which is made of transparent plexiglass material with dimensions of 40 × 40 × 31 cm, was used to evaluate spontaneous locomotor activities of the rats in the experimental groups. Locomotor activities of the animals in the vertical and horizontal directions were recorded for 10 min [54,55].

### 4.7. Statistical Analysis

For statistical calculations, Graphpad Prism. 6.01 package program was used. Data acquired from the Randall-Selitto, Hargreaves and activity cage tests, which were conducted in the same groups for consequent weeks, were analyzed by two-way repeated ANOVA, followed by Bonferroni tests as post hoc. Besides data obtained from the mechanistic studies were analyzed by two-way ANOVA, followed by the same multiple comparison tests.

Results were expressed as mean ± standard error of mean (S.E.M.). *p* value of 0.05 was considered as significant.

## Figures and Tables

**Table 1:** Experimental groups.

Experimental Groups	Treatment	
	Day 0	Days 28–42
Control group	Citrate buffer, i.v.	Physiological saline, p.o.
DM group	STZ, i.v.	Physiological saline, p.o.
Pregabalin + DM group	STZ, i.v.	Pregabalin (10 mg/kg, p.o) [45,46]
Atomoxetine + DM group	STZ, i.v.	Atomoxetine (3 mg/kg, i.p.) [47]
